# PMA-qPCR method for the selective quantitation of viable lactic acid bacteria in fermented milk

**DOI:** 10.3389/fmicb.2022.984506

**Published:** 2022-09-07

**Authors:** Zihang Shi, Xiefei Li, Xiankang Fan, Jue Xu, Qing Liu, Zhen Wu, Daodong Pan

**Affiliations:** ^1^State Key Laboratory for Managing Biotic and Chemical Threats to the Quality and Safety of Agro-Products, Ningbo University, Ningbo, China; ^2^Key Laboratory of Animal Protein Food Processing Technology of Zhejiang Province, College of Food and Pharmaceutical Sciences, Ningbo University, Ningbo, China; ^3^Nanjing Dairy Group, Nanjing, China

**Keywords:** lactic acid bacteria, *pheS* gene, PMA-qPCR, viable bacteria numbers, fermented milk

## Abstract

The number of viable lactic acid bacteria (LAB) is a key indicator of the quality of fermented milk. Currently, the combination of propidium monoazide (PMA) and qPCR has been applied in the quantification of viable bacteria in various matrices. In this research, the PMA-qPCR method was used to detect the number of viable bacteria of each LAB species in fermented milk. By analyzing *pheS* gene and 16S rRNA gene sequence similarities in five species of LAB, namely *Lactobacillus delbrueckii* subsp. *bulgaricus*, *Lactiplantibacillus plantarum*, *Streptococcus thermophilus*, *Lactobacillus helveticus*, and *Lactococcus lactis* subsp. *lactis*, the *pheS* gene resolved species identities better and was thus selected to design specific primers and probes. The *pheS* gene was cloned into the pUC19 vector and used to construct a standard curve for absolute quantification. Standard curves for quantification were constructed for each LAB species for serial dilutions between 10^11^ and 10^6^ CFU/mL, with *R*^2^ > 0.99. The number of viable bacteria in the fermented milk detected by PMA-qPCR was significantly lower than that of qPCR (*P* < 0.05), indicating that PMA inhibited the amplification of DNA from dead cells. This was corroborated by the results from bacterial staining and plate count experiments. The proposed PMA-qPCR method provided rapid qualitative and quantitative determination of the number of viable bacteria for each LAB species in fermented milk within 3 h.

## Introduction

Fermentation is an ancient food processing technique that has been used worldwide for thousands of years (Tamang et al., 2020). Fermented milk is a typical fermented food, it is produced by fermenting milk with lactic acid bacteria (LAB), LAB strains are usually isolated from nature and can survive in the gastrointestinal tract (Rai and Tamang, 2022). Fermented milk is very popular among consumers because of its unique taste, good flavor, and rich nutritional content (Benozzi et al., 2015; Morell et al., 2015; Aryana and Olson, 2017). Besides these, the ingestion of fermented milk can also promote health, for example, consuming Kefir fermented milk was considered to be an adjuvant therapy for the prevention of diabetes (Azizi et al., 2021). There are now more than 500 fermented milk products on the market and the number continues to increase. The quality of fermented milk is influenced by the number of viable LAB bacteria, and the viable count of LAB in fermented milk should not fall below 10^6^ CFU/mL throughout its shelf life (FAO/WHO, 2011). However, various LAB species are cultivated under similar conditions, impeding the quick and accurate determination of the viable count of each species *via* the cultivation method (Süle et al., 2014). Thus, there is a need to develop a new method that can quickly determine the number of viable bacteria for each LAB species in fermented milk.

Nascent techniques for the detection of bacteria under non-culture conditions include flow cytometry, qPCR, 16S rRNA gene sequencing, and metagenomic (Paparella et al., 2008; Cao et al., 2019; Biçer et al., 2021; Nakibapher Jones Shangpliang and Tamang, 2021). In 2015, flow cytometry was recommended by the International Organization for Standardization (ISO) as a standard method for probiotic counting (ISO, 2015). It can be combined with fluorescent dyes such as SYTO9 and propidium iodide (PI) for rapid detection of viable and dead cells in the sample (Pane et al., 2018). It is also suitable for the identification of bacterial species in a sample with the use of special antibody with fluorescent markers combined with PI dye, but this method is expensive (Wang et al., 2020). A common method for determining the number of microorganisms in food is qPCR, and it has the advantages of short testing time, high level of accuracy, and low cost (Yoon et al., 2018; Kim et al., 2020). In bacteria, the 16S rRNA gene is often used for species identification, but it does not discriminate between closely related species, necessitating the use of protein-encoding housekeeping genes that have higher rates of evolution (Lee et al., 2015; Bottari et al., 2017).

The inability to discriminate between viable and dead cells is a major disadvantage of the qPCR method. In recent years, nucleic acid binding dyes, such as propidium monoazide (PMA) have been used to overcome this challenge (Fittipaldi et al., 2012; Porcellato et al., 2015). PMA is a membrane-permeant that selectively enters membrane-damaged cells and irreversibly binds to DNA in the presence of light, which prevents its PCR amplification. Combining this with qPCR can quickly and accurately determine the number of viable bacteria in the sample. At present, this method has been applied in the detection of several species, such as *Listeria monocytogenes* and *Lacticaseibacillus paracasei* (Scariot et al., 2018; Kragh et al., 2020). However, current studies have been limited to the determination of the viable number of a single bacterial species in samples by PMA-qPCR.

In this study, we developed a method that could rapidly detect and quantify different LAB specie in fermented milk. *L. plantarum* A3, *L. helveticus* CGMCC 1.9090, and *Lactococcus lactis* subsp. *lactis* CGMCC 1.1936 (*L. lactis*) were used with yogurt starter (*Lactobacillus delbrueckii* subsp. *bulgaricus* and *Streptococcus thermophilus*) to produce fermented milk. Whether the phenylalanine-tRNA ligase subunit alpha (*pheS*) gene could be used as a new marker for the identification of LAB species was verified. The specific primers and probes for the *pheS* gene were also designed to determine the number of multiple viable LAB in fermented milk by PMA-qPCR. The method was validated by comparing results with those of the plate count method, qPCR, and confocal laser scanning microscopy.

## Materials and methods

### Bacterial strains and culture conditions

*Lactobacillus delbrueckii* subsp. *bulgaricus* KLDS1.0207, *L. helveticus* CGMCC 1.9090, *S. thermophilus* ABT-T, *L. plantarum* A3, *L*. *lactis* CGMCC 1.1936 were obtained from the China General Microbiological Culture Collection Center (CGMCC, Beijing, China). *S. thermophilus* was cultivated in M17 liquid medium at 42°C and *L. lactis* was cultivated in M17 liquid medium at 30°C. *L. delbrueckii* subsp. *bulgaricus*, *L. plantarum*, and *L. helveticus* were cultivated in MRS liquid medium at 37°C.

### Sequence similarity analysis of *pheS* and 16S rRNA genes in five species of lactic acid bacteria

The sequences of the 16S rRNA gene and the *pheS* gene of the type strain in each LAB were downloaded from the NCBI database,^1^ which included *L. delbrueckii* subsp. *bulgaricus* ATCC 11842, *S. thermophilus* ATCC 19258, *L. helveticus* DSM 20075, *L. lactis* JCM 5805, and *L. plantarum* ATCC 14917. The sequence alignment function of the Geneious prime software^2^ was used to analyze sequence similarity between the *pheS* gene and the 16S rRNA gene.

### Construction of pUC19-*pheS* recombinant plasmid

The DNA samples were extracted from 3 mL of bacterial cultures of LAB with a TIANamp Bacteria DNA kit (Tiangen Biotech Co., Ltd., Beijing, China) according to the manufacturer’s instructions. Cloning primers with homology arms were designed by CE design software (Vazyme Biotech Co., Ltd., Nanjing, China) (Table 1). The PCR reaction components included 25°μL of PrimeSTAR Max DNA Polymerase (Takara Biomedical Technology (Beijing) Co., Ltd., Beijing, China), 2°μL of each primer (10°μmol/L), 2°μL of DNA template, and 19°μL of RNase free ddH_2_O. The PCR reaction procedure was as follows: 95°C for 5 min, followed by 30 cycles of 95°C for 30 s, 58°C for 30 s, 72°C for 1 min, and finally 72°C for 10 min, 4°C for 30 min. The PCR product was purified with an E.Z.N.A.^®^ Gel Extraction Kit (Omega Bio-tek Inc., Norcross, GA, United States). The pUC19 plasmid was linearized by *SmaI* and *HindIII* restriction endonucleases [Takara Biomedical Technology (Beijing) Co., Ltd., Beijing, China]. The plasmid linearization reaction components included 1°μg of pUC19 plasmid, 1°μL each of *SmaI* and *HindIII* endonucleases, 2°μL of 10 × quickcut green buffer, and supplemented with ddH_2_O to a total volume of 20°μL. The plasmid linearization procedure was as follows: 37°C for 20 min, 85°C for 20 min, and 4°C for 30 min. The PCR purification product and linearized pUC19 plasmids were ligated using the ClonExpress Ultra One Step Cloning Kit (Vazyme Biotech Co., Ltd., Nanjing, China). The recombinant plasmid was sequenced using the M13 universal primer to confirm the correct ligation and then extracted with a HiPure Plasmid Plus Micro Kit (Magen Biotech Co., Ltd., Guangzhou, China) according to the manufacturer’s instructions. Plasmid concentrations were determined using a NanoDrop ND-2000 spectrophotometer [Thermo Fisher Scientific (Shanghai), Co., Ltd., Shanghai, China].

**TABLE 1:** Recombinant plasmid ligation primers sequences for the *pheS* gene.

Species	Primers	Sequence (5′-3′)	Position (5′-3′)	GenBank ID
*L. delbrueckii* subsp. *bulgaricus*	forward	aattcgagctcggtacccgggATGGATTTATTTGATCGATTGAAGG	1–25	CP041280.1
	reverse	gaccatgattacgccaagcttTTAATCTTCCTCCTGACGGAATTG	1027–1050	
*S. thermophilus*	forward	aattcgagctcggtacccgggATGGATTTACAAACACAATTACAAGAGTT	1–29	NC017581.1
	reverse	gaccatgattacgccaagcttTTATTTAAACTGTTCTGAGAATCGAACG	1017–1044	
*L. helveticus*	forward	aattcgagctcggtacccgggATGGACTTATTTGATAAGTTAAAAGAGCTT	1–30	CP003799.1
	reverse	gaccatgattacgccaagcttTTAATTTTCCTCCTTGCGGAAT	1029–1050	
*L. plantarum*	forward	aattcgagctcggtacccgggATGAGTTTACAAGATCGATTAACCGA	1–26	NC014554.1
	reverse	gaccatgattacgccaagcttCTAACCTTTCTTATAGAACTGTGACAAGAA	1018–1047	
*L. lactis*	forward	aattcgagctcggtacccgggATGAACTTACAAGAAAAAATTGAAGACC	1–28	CP065737.1
	reverse	gaccatgattacgccaagcttTTATTTTCCAAATTGCTCTAAGAATCG	1015–1041	

### Construction of standard curves

Primers and probes targeting the *pheS* gene in five LAB were designed using the Geneious prime software (Table 2). The primer-blast database from NCBI^3^ was used to check primer specificity. The specificity of the primers was verified through the PCR and the PCR reaction conditions as described in section “Construction of pUC19-*pheS* recombinant plasmid.” The plasmid copy number was determined by the formula:


$$\text{Plasmid copy}\,\mathrm{number}\,\left(\mathrm{copies}/\mu \,L\right)=\frac{\mathrm{DNA}\,\mathrm{concentration}\,\left(\mathrm{ng}/\mu \,L\right)\times 10^{-9}}{\mathrm{fragment}\,\mathrm{size}\times 660}\times \text{NA}$$

**TABLE 2:** Specific primers and probes sequences for the *pheS* gene.

Species	Primers and probes	Sequence (5′-3′)	Position (5′-3′)	GenBank ID
*L. delbrueckii* subsp. *bulgaricus*	forward	CGGGACATGCAGGCTACTTT	463–482	
	reverse	GAGATGTTCTTGCCGACAACCA	656–677	CP041280.1
	probe	FAM-CACCTGCTGCGGAGCCAGAC-BHQ1	499–518	
*S. thermophilus*	forward	TGGCTGAAATGCGTGGTGAACA	53–74	
	reverse	TCCCTTCAAAAGTTCCGTCAAAGAACC	121–147	NC017581.1
	probe	FAM-CAAGAATTGCGTGTTGCAGTTTTAGGT-BHQ1	88–114	
*L. helveticus*	forward	TGACGATGCTACTCACTCACACCA	612–635	
	reverse	ACTTGGACGAAGACGAGTAGCTCTAT	734–759	CP003799.1
	probe	FAM-TGCCAAGCACGTCTTCGGTCA-BHQ1	711–731	
*L. plantarum*	forward	CGGATTCAGGCTGCGATTG	205–223	
	reverse	GGCTGACCTTGTGGAACTTC	307–326	NC014554.1
	probe	FAM-CGTGACGTTACCGGGTCGGG-BHQ1	288–307	
*L. lactis*	forward	TGAAGACCTTCGCAAGCGGACT	21–42	
	reverse	AAGCATTGGCAAGTGCGCCA	177–196	CP065737.1
	probe	FAM-ACTGTGATGCTCGGTAAAAAGGGTG-BHQ1	94–118	

where: NA is Avogadro’s constant, NA = 6.02 × 10^23^, 660 is the average molecular weight of a base in double-stranded DNA.

The recombinant plasmids were diluted 10-fold in RNase free ddH_2_O to achieve final copy number between 10^6^ and 10^11^ copies per reaction. qPCR reactions were performed using a Roche Light Cycler 96 System (Roche Molecular Systems, Inc., Basel, Switzerland). According to the reaction conditions of the 2 × Spark Universal Depolluting Probe qPCR Mix protocol (Sparkjade Biotech, Co., Ltd., Shandong, China), the total reaction volume was 25°μL: 12.5°μL of 2 × Spark Universal Depolluting Probe qPCR Mix, 0.5°μL of each forward and reverse primer (10°μmol/L), 0.25°uL of probe (10°μmol/L), 1°μL of DNA template, 10.25°μL of RNase free ddH_2_O. The cycling conditions were: 2 min at 37°C, 5 min at 95°C, followed by 45 cycles of denaturation at 95°C for 10°s and annealing at 60°C for 30°s. The results were analyzed using Light 96 software (Roche Molecular Systems, Inc., Basel, Switzerland). The following equation was used to calculate the amplification efficiency during quantitative PCR:


$$\text{E}=10^{-1/\text{slope}}-1$$

where: E is the amplification efficiency, and the slope is the slope of the standard curve.

### Preparation of fermented milk

Skimmed milk powder^®^ (Maxigences Skimmed Instant Milk Powder, Maxigences Pty Ltd., Sydney, Australia) (12.5%, w/v) and sucrose (3%, w/v) were dissolved in distilled water (84.5%, w/v) at 60°C. The milk was homogenized for 20 min at 6,000 rpm using a high-speed disperser (XHF-D, Xinzhi Corp., Ningbo, China). The homogenized milk was heat treated at 95°C for 10 min and cooled to 42°C. Samples were collected from the 16-h LAB culture and centrifuged at 8,000 *g* for 10 min. The supernatant was discarded, and bacteria were washed three times with sterile saline. The strain combinations for the subsequent fermenting of milk were as follows: *L. delbrueckii* subsp. *bulgaricus* + *S. thermophilus* + *L. helveticus* + *L. plantarum* (group A), *L. delbrueckii* subsp. *bulgaricus* + *S. thermophilus* + *L. plantarum* + *L. lactis* (group B), *L. delbrueckii* subsp. *bulgaricus* + *S. thermophilus*+ *L. lactis* + *L. helveticus* (group C), *L. delbrueckii* subsp. *bulgaricus* +*S. thermophilus* + *L. helveticus* + *L. plantarum* + *L. lactis* (group D). The initial viable cell density of each LAB inoculum was 5 × 10^6^ CFU/mL. The fermentation was done at 42°C for 7 h, and after completion, fermented milk was stored at 4°C for 16 h.

### Lactic acid bacteria viable bacteria quantification by PMA-qPCR

Following the manufacturer’s instructions, 1 mg PMA (Biotium Inc., Hayward, CA, United States) was dissolved in 20% dimethyl sulfoxide (DMSO) to obtain a 20°mmol/L stock solution and stored at −20°C in the dark. PMA solution (2.5°μL) was added to 1 mL fermented milk to a final concentration of 50°μmol/L. The samples were mixed once every minute for 10 min in the dark, then it was diluted 100 times with sterile saline and exposed for 15 min under a 100°W LED light source, while on ice to prevent heating during the exposure. After PMA treatment, DNA was extracted from 2 mL of the diluted sample using a TIANamp Bacteria DNA kit (Tiangen Biotech Co., Ltd., Beijing, China). The DNA samples were subjected to qPCR reactions under the aforementioned conditions. The formula shown below was used to calculate the number of viable bacteria in an initial 1 mL of fermented milk:


Number of viable bacteria=[(Cqvalue-intercept)/slope]×50×50

where: the slope and intercept used in the formula are the same as those used in the standard curve, the first 50 is the total volume of DNA extracted from the 2 mL diluted sample, and the second 50 is the dilution multiple.

### Detection of viable bacteria in fermented milk by qPCR and plate count

One mL of non-PMA treated fermented milk was diluted 100 times, and 2 mL dilution was aspirated for DNA extraction. DNA was used for qPCR experiments under the aforementioned conditions. Milk not inoculated with LAB was used as a negative control.

The number of viable bacteria in the fermented milk was determined under different conditions. Modified reinforced clostridial media with aniline blue dye was used for selective identification of *L. delbrueckii* subsp. *bulgaricus* (Nwamaioha and Ibrahim, 2018). The SPY9.3 selective medium was used to determine the number of viable *S. thermophilus* bacteria in fermented milk (Shani et al., 2021). *L. helveticus* counts were determined by cultivation on MRS agar incubated for 48 h at 43°C (Cuffia et al., 2020). MRS agar supplemented with 10 mg/L vancomycin was used to identify the number of *L. plantarum* (Li et al., 2017). The M17 and Reddy’s agars were used to detect *L. lactis* (Poudel et al., 2022). As these methods do not accurately discriminate between LAB species, single colonies were picked from plates, and processed with the Lysis Buffer for Microorganism to Direct PCR kit [Takara Biomedical Technology (Beijing) Co., Ltd., Beijing, China], then used in PCR reactions, the primers used for the PCR were shown in Table 2.

### Bacterial staining for viable and dead bacteria in fermented milk

The fermented milk was diluted 100 times with sterile water, and the samples were processed with a LIVE/DEAD™ BacLight™ Bacterial Viability Kit [Thermo Fisher Scientific (Shanghai), Co., Ltd., Shanghai, China]. SYTO9 and PI were mixed in a 1:1 ratio in a brown 1.5 mL tube with a pipette, then 3°μL of the dye mixture was added to 1 mL of the diluted sample, mixed well, and incubated in the dark for 15 min at room temperature. Subsequently, 5°μL stained sample was dropped on a glass slide, covered with a coverslip. The stained LAB was observed and photographed under an LSM880 confocal laser scanning microscope (Carl Zeiss AG., Oberkochen, Germany).

### Statistical analysis

Three independent replicates of each sample were used in the experiments. The experimental data were analyzed by SPSS software (SPSS Inc., Chicago, IL, United States), and one-way analysis of variance (ANOVA) was used to compare differences between the results obtained by PMA-qPCR, qPCR, and plate count. *P* < 0.05 was considered statistically significant. Graphs were constructed using Origin 2018 software (Origin Lab Corp., Northampton, MA, United States).

## Results

### Comparison of sequence similarity of the *pheS* and 16S rRNA gene

**FIGURE 1:**
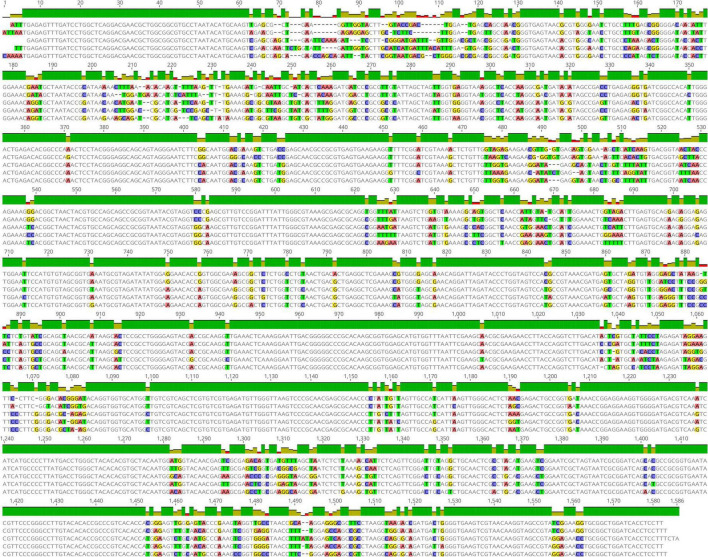
Multiple sequence alignment of the 16S rRNA gene of five lactic acid bacteria (LAB) type strains, namely *Lactobacillus delbrueckii* subsp. *bulgaricus* ATCC 11842, *Streptococcus thermophilus* ATCC 19258, *Lactobacillus helveticus* DSM 20075, *Lactococcus lactis* JCM 5805, and *Lactiplantibacillus plantarum* ATCC 14917. Positions with differential bases were marked by a different color.

**FIGURE 2:**
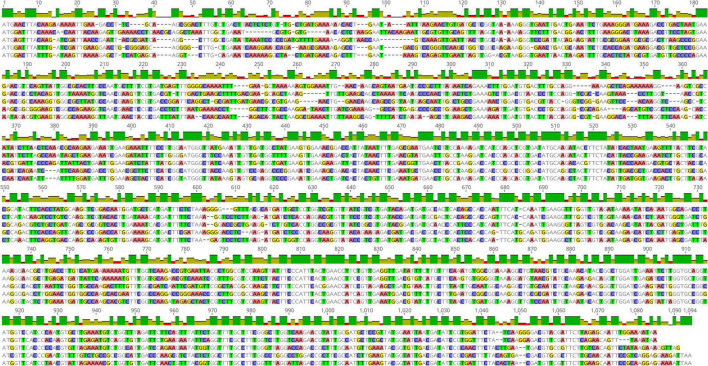
Multiple sequence alignment of the *pheS* gene of five lactic acid bacteria (LAB) type strains, namely *Lactobacillus delbrueckii* subsp. *bulgaricus* ATCC 11842, *Streptococcus thermophilus* ATCC 19258, *Lactobacillus helveticus* DSM 20075, *Lactococcus lactis* JCM 5805, and *Lactiplantibacillus plantarum* ATCC 14917.

The multiple sequence alignment of the *pheS* and 16S rRNA gene of the five LAB species are shown in Figures 1, 2. The sequence similarity of the *pheS* gene was much lower compared with that of the 16S rRNA gene. The 16S rRNA of the five LAB species was 1,586 bp in length, in which 1,208 bases were identical, accounting for 76.3% of the full length of the sequence. The *pheS* gene was 1,094 bp in length, with 439 bases of identical sequence, which accounted for 40.1% of the full length. The lower sequence similarity allowed us to design specific primers and probes for each species, so the *pheS* gene was selected as the target gene for the identification of LAB species.

### Primer specificity validation and analysis of standard curves

**FIGURE 3:**
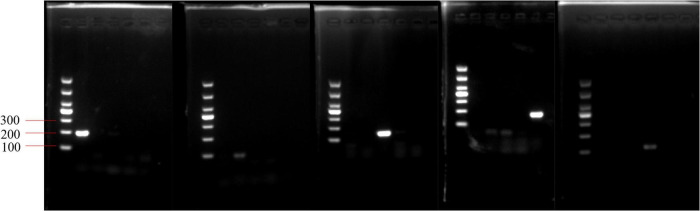
Validation of primer specificity for the *pheS* gene. From left to right, the order of the primers in the five electropherograms is *Lactobacillus delbrueckii* subsp. *bulgaricus*, *Streptococcus thermophilus*, *Lactobacillus helveticus*, *Lactococcus lactis*, and *Lactiplantibacillus plantarum*. In each electropherogram, the sequence of DNA templates is *L. delbrueckii* subsp. *bulgaricus*, *S. thermophilus*, *L. helveticus*, *L. plantarum*, and *L. lactis*.

Key to the success of the PMA-qPCR experiment was the specificity of the primers. The PCR assay was performed to validate primer specificity. Each pair of primers only amplified the target fragment from the targeted strain, amplicons produced single and bright bands of the expected length (100–200 bp) and lacked spurious products (Figure 3).

**FIGURE 4:**
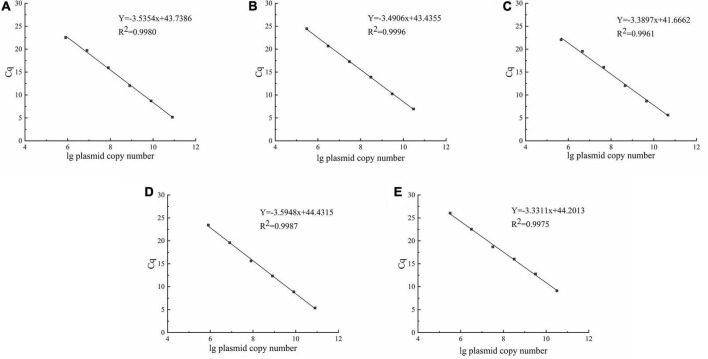
Standard curves were constructed using the pUC19-*pheS* recombinant plasmid as reaction template. **(A–E)** represent *Lactobacillus delbrueckii* subsp. *bulgaricus*, *Streptococcus thermophilus*, *Lactobacillus helveticus*, *Lactococcus lactis*, and *Lactiplantibacillus plantarum*, respectively.

The standard curves for each LAB were established with the pUC19-*pheS* recombinant plasmid as the reaction template. There was a good linear fit between Cq values and lg plasmid copy number (Figure 4). The correlation coefficients for all standard curves exceeded 0.99. The amplification efficiencies of the primers used in this study were *E* = 91.8% for *L. delbrueckii* subsp. *bulgaricus*, *E* = 93.41% for *S. thermophilus*, *E* = 97.25% for *L. helveticus*, *E* = 89.75% for *L. lactis*, and *E* = 99.62% for *L. plantarum.*

### Detection of viable bacteria in fermented milk by PMA-qPCR, qPCR, and plate count

**FIGURE 5:**
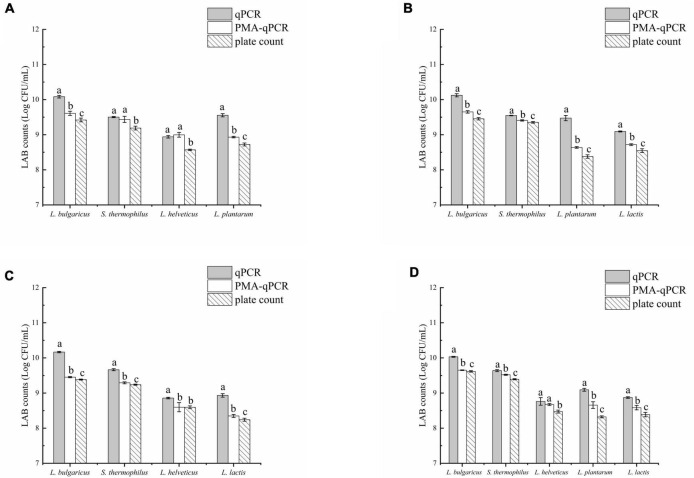
qPCR, PMA-qPCR, and plate count were used to determine the number of viable lactic acid bacteria (LAB) in fermented milk. **(A)** *Lactobacillus delbrueckii* subsp. *bulgaricus* + *Streptococcus thermophilus* + *Lactobacillus helveticus*+ *Lactiplantibacillus plantarum*, **(B)** *L. delbrueckii* subsp. *bulgaricus* + *S. thermophilus*+ *L. plantarum* + *Lactococcus lactis*, **(C)** *L. delbrueckii* subsp. *bulgaricus* + *S. thermophilus* + *L. lactis*+ *L. helveticus*, and **(D)** *L. delbrueckii* subsp. *bulgaricus* + *S. thermophilus* + *L. helveticus*+ *L. plantarum + L. lactis.* The letters (a,b, and c) were used to indicate the difference between the results of the three methods. *p* < 0.05 was considered to be a significant difference.

The results of the three methods used to detect viable bacteria in group A are shown (Figure 5A). The viable counts of *L. delbrueckii* subsp. *bulgaricus* and *L. plantarum* significantly differed among all three methods (*p* < 0.05), whereas those of *S. thermophilus* and *L. helveticus* as detected by qPCR and PMA-qPCR did not significantly differ (*p* > 0.05) but were both significantly higher than counts obtained by the plate count (*p* < 0.05). Figure 5B showed the results of the viable bacteria determination experiment for group B, it was found that there was a significant difference between the results of all three methods (*p* < 0.05). The counts determined by the PMA-qPCR method were fewer than those from the qPCR method but higher than from the plate count. In group C, *L. delbrueckii* subsp. *bulgaricus*, *S. thermophilus*, and *L. lactis* counts obtained by PMA-qPCR were significantly lower than from qPCR but higher than those obtained from the plate count (*p* < 0.05). However, for *L. helveticus*, counts from PMA-qPCR and plate count were similar and both were significantly lower than those from qPCR. In Figure 5D, the experimental results for *L. delbrueckii* subsp. *bulgaricus*, *S. thermophilus*, *L. plantarum*, and *L. lactis* all differed significantly due to different methods. The results for *L. helveticus* viable counts by qPCR and PMA-qPCR were not significantly different but both were higher than the plate count (*p* < 0.05).

### Detection of viable bacteria in fermented milk by confocal laser scanning microscope

**FIGURE 6:**
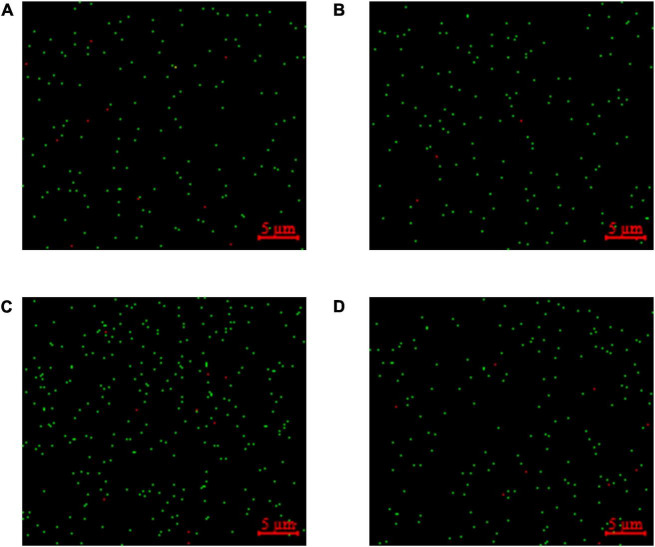
Staining for viable and dead bacteria in fermented milk. **(A)** *Lactobacillus delbrueckii* subsp. *bulgaricus* + *Streptococcus thermophilus* + *Lactobacillus helveticus*+ *Lactiplantibacillus plantarum*, **(B)** *L. delbrueckii* subsp. *bulgaricus* + *S. thermophilus*+ *L. plantarum* + *Lactococcus lactis*, **(C)** *L. delbrueckii* subsp. *bulgaricus* + *S. thermophilus* + *L. lactis*+ *L. helveticus*, and **(D)** *L. delbrueckii* subsp. *bulgaricus* + *S. thermophilus* + *L. helveticus*+ *L. plantarum + L. lactis.* The viable bacteria emit green fluorescence and the dead bacteria emit red fluorescence.

The LIVE/DEAD™ BacLight™ Bacterial Viability Kit utilized both SYTO 9 green-fluorescent nucleic acid and PI red-fluorescent nucleic acid dyes. The viable bacteria in the sample are stained green, while the dead bacteria are stained red. The percentages of viable bacteria in samples of groups A-D were 93.90, 97.41, 95.10, and 96.78%, respectively (Figures 6A–D).

## Discussion

PMA-qPCR has been successfully applied to various fermented foods to detect the number of viable microbial cells, and it is a viable way to genetically differentiate species. For species not easily discriminated and selected on culture media, it is important to select the right target gene to design specific primers. The target gene should ideally be present in single copy form, evolve more rapidly than rRNA genes, and be widely distributed throughout the bacterial genus. Moreover, the target gene should provide sufficient information, due to its inherent variability, to distinguish species of a particular genus (Zeigler, 2003). The *pheS* and RNA polymerase alpha subunit (*rpoA*) housekeeping genes have sufficient robustness and variability for the identification of all recognized species of the genus *Enterococcus* (Stackebrandt et al., 2002; Santos and Ochman, 2004; Naser et al., 2005). Multiple sequence analyses of *pheS* and *rpoA* genes from *Lactobacillus* revealed that the *pheS* gene sequences had an interspecies gap, which in most cases exceeded 10% dissimilarity and an intraspecies dissimilarity of up to 3%. For the *rpoA* gene sequences, interspecies dissimilarities typically exceeded 5% and intraspecies dissimilarities were up to 2% (Naser et al., 2007). On the other hand, by comparing the sequence similarity of the 16S rRNA genes of the five LAB species, it was found that they were too similar to design specific primers. The copy number of the 16S rRNA gene also varies in different species of LAB (Lee et al., 2008). Thus, the *pheS* gene was selected for our nascent method for the identification of LAB species in fermented milk.

The qPCR experiment requires an accurate standard curve that is not influenced by the food matrix, and the use of recombinant plasmids as qPCR reaction templates eradicates this influence (Rizzotti et al., 2015). Although bacterium genomic DNA as a template for qPCR reactions has been used, it resulted in lowly accurate standard curves (Yang et al., 2021). This was because LAB genomes differ in size, even within the same species. For example, the genomes for *L. plantarum* ST-III and *L. plantarum* WCFS1 are 3.308 and 3.349°Mbp respectively—this can lead to errors in the calculation of copy numbers. Conversely, the molecular weight of the recombinant plasmid was accurately calculated using a known formula and the number of its bases. This molecular weight of the plasmid was much lower than that of genomic DNA, allowing for easy adjustment of the standard curve detection range. A reliable standard curve R^2^ should be close to 1, with a slope between −3.9 and −3.0 and PCR amplification efficiency of 80–115% (Zhang and Fang, 2006). Each standard curve met the requirements and was used for data analysis. Globally, most countries generally require a microbial count of more than 10^6^ CFU/mL for the sale of fermented milk products (FAO/WHO, 2011). Thus, the qPCR standard curves constructed in this study fully met this requirement.

The qPCR method detects the total number of bacteria in the fermented milk whereas the PMA-qPCR method detects only that of viable bacteria. By comparing the results from PMA-qPCR to those of the qPCR method, PMA eliminated the qPCR signal of dead cells in fermented milk, resulting in a more accurate number of viable bacteria in the sample. A similar approach was used to quantify the viable counts of *L. paracasei* in fermented milk (Scariot et al., 2018). In their study, the elongation factor Tu (*tuf*) gene in *L. paracasei* was selected as the target gene to design specific primers and the results showed that the viable counts of *L. paracasei* obtained by PMA-qPCR were significantly lower than those obtained by qPCR. Comparing the results of the PMA-qPCR and the plate count, the results of the plate count were lower than the PMA-qPCR method, probably because some of the cells in the samples were in a viable but non-culturable (VBNC) state. Bacteria in this self-protective state cannot be detected by the plate count method, because they cannot grow into visible colonies on the plate. However, PMA-qPCR can successfully detect VBNC bacteria in samples, for example, Liu et al. (2018) successfully detected the cell number of *Vibrio parahaemolyticus* in the VBNC state using PMA-qPCR. The bacterial staining experiments were also conducted to assess the inhibitory effect of PMA on the amplification of DNA from dead bacteria in the samples. The LIVE/DEAD™ BacLight™ Bacterial Viability Kit was used to detect viable and dead LAB in fermented foods (Fernández-Pérez et al., 2019). The results of the bacterial staining experiments showed that the percentage of viable bacteria in all samples was above 90%, which corroborated experimental results of the PMA-qPCR method, and thus supported the hypothesis that PMA effectively bound covalently to DNA in dead bacteria, ensuring that only viable were detected by our PMA-qPCR method.

## Conclusion

In this study, we analyzed multiple sequence alignments of the *pheS* and 16S rRNA genes of LAB and demonstrated the *pheS* gene was more effective for molecular identification of phylogenetically closely related species. We developed a method for rapid detection of the number of viable bacteria of each LAB species in fermented milk, the entire experiment took no more than 3 h. Using counts obtained from the plate count method coupled with bacterial staining experiments, we demonstrated that the proposed PMA-qPCR method detected the number of viable bacteria in fermented milk with high accuracy. However, PMA-qPCR does not detect the number of viable bacteria of unknown bacterial species in the sample. This method holds great application potential in the detection of several viable bacteria in other fermented foods. At the same time, we will also develop universal primers or probes to make up for the disadvantage that PMA-qPCR cannot detect the number of viable bacteria of unknown species in the sample.

## Data availability statement

The datasets presented in this study can be found in online repositories. The names of the repository/repositories and accession number(s) can be found in the article/supplementary material.

## Author contributions

ZS: conceptualization, methodology, data curation, formal analysis, writing-original draft, and writing-review and editing. XL: conceptualization and methodology. XF: methodology, software, and data curation. JX: software and validation. DP: conceptualization, project administration, funding acquisition, validation, supervision, and writing-review and editing. ZW: supervision, validation, visualization, and resources. QL: visualization and investigation. All authors contributed to the article and approved the submitted version.

## Footnotes

1
https://www.ncbi.nlm.nih.gov/

2
http://www.geneious.com

3
https://www.ncbi.nlm.nih.gov/tools/primer-blast/index.cgi?LINK_LOC=BlastHome
